# The impact of Qodume Shirazi seed mucilage‐based edible coating containing lavender essential oil on the quality enhancement and shelf life improvement of fresh ostrich meat: An experimental and modeling study

**DOI:** 10.1002/fsn3.1940

**Published:** 2020-10-13

**Authors:** Samaneh Heydari, Hossein Jooyandeh, Behrooz Alizadeh Behbahani, Mohammad Noshad

**Affiliations:** ^1^ Department of Food Science and Technology Faculty of Animal Science and Food Technology Agricultural Sciences and Natural Resources University of Khuzestan Mollasani Iran

**Keywords:** antimicrobial activity, chemical composition, edible coating, modeling, natural preservative

## Abstract

Today, food consumers prefer to use the foods that contain natural preservatives. The purpose of this study was to investigate the effect of Qodume Shirazi seed mucilage (QSSM) and lavender essential oil (LO) on the preservation of ostrich meat during cold storage. The chemical compounds of LO were identified through gas chromatography–mass spectrometry (GC/MS). The ostrich meat samples were coated with the mucilage containing the essential oil at concentrations of 0%, 0.5%, 1%, 1.5%, and 2%, v/v. The control and the coated ostrich meat samples were kept at 4°C and analyzed for microbiological (total viable count, psychrotrophic count, *Escherichia coli*, *Staphylococcus aureus*, coliforms, and fungi), physicochemical (moisture content, pH, texture, and color parameters), and sensorial (odor, color, and total acceptance) characteristics during 9 days of storage. GC/MS identified 12 compounds in the essential oil, among which linalool was the major one (43.3%). The lightness (L* value) and hardness of all the ostrich meat samples were reduced during the storage. From a microbiological point of view, the cold storage duration for the control and the coated sample without the essential oil was only 3 days, while for coated samples containing 0.5%, 1%, 1.5%, and 2% essential oil, it was 3, 3, 6, and 9 days, respectively. The coated ostrich meat containing 2% LO had an appropriate quality with an expanded shelf life. The results showed that neural network with 10 neurons in the hidden layer had the lowest mean squared error and mean absolute error and the highest correlation coefficient for predicting the quality and microbial properties of the coated meat samples during storage.

## Graphical abstract

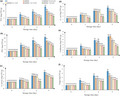

## 1 INTRODUCTION

Ostrich meat is considered as a healthy alternative to other red meats in Western societies due to its favorable nutritional properties and one of the most popular sources of protein (low cholesterol and intramuscular fat contents and generally high omega‐3 polyunsaturated fatty acids (PUFA x3) percentages). In recent years, there is an increasing trend toward consumption of ostrich meat. Therefore, improving the quality and increasing the shelf life of this product are very important both for local consumption and for export. Much research has been done to prevent microbial growth and sensory properties of ostrich meat. Factors such as initial microbial loads, packaging atmosphere, pH, storage temperature (and temperature abuse), and sampling day could affect the quality of ostrich meat (González‐Montalvo et al., 2007). For example, the relatively high pH of ostrich meat is one of the factors that can cause rapid microbial spoilage in some packaging conditions. Another factor that may cause ostrich meat spoilage is its the higher polyunsaturated fatty acid content as compared to beef and chicken meats, which makes ostrich meat to be more sensitive to oxidative deterioration (Divani et al., 2017).

Modern packaging technologies can increase the shelf life of a packaged product by preventing or delaying microbial growth. This can be achieved by the manipulation of the meat microenvironment (González‐Montalvo et al., 2007).

Due to the abuse of some chemical additives and preservatives and their harmful effects on human health, many countries have regulated food production. For this reason, interest in studying and researching on natural preservatives and replacing chemical preservatives with natural ones has been extensively increased. In addition, the excessive use of chemical preservatives leads to the development of bacterial resistance against antibiotics. Therefore, the study of natural antimicrobials has also received medical attention (Gonçalves Cattelan et al., 2018).

On the other hand, in addition to the growing demands of consumers to achieve a food with the high level of quality, environmental concerns about the negative impact of plastic packaging residues have recently attained more attention. Therefore, food industry experts try to create the new type of edible films and coatings to preserve food quality.

Edible films and coatings that are named and classified based on the compounds used contain different types of polysaccharides, lipids, proteins, or their combinations. The use of edible films/coatings as food packaging has many advantages, including environmental friendliness, economic viability, long shelf life, and good barrier properties for gases and carriers of other food additives such as antimicrobial, vitamins, and antioxidant agents (Hashemi & Mousavi Khaneghah, 2017).

Edible coatings are edible films made on the exterior surfaces of a food or material (Jooyandeh, 2011). In fact, edible coatings form a thin layer on fresh foods such as fruits, vegetables, and meat, thereby maintaining postharvest quality and increasing crop shelf life. The use of food coatings is a relatively simple, environmentally friendly, and inexpensive technology to reduce the spoilage of fruits and vegetables, and therefore has been relatively more accepted by consumers as compared to other storage methods such as the use of chemical preservatives or radiation (Saha et al., 2017).

Chemical composition and applications of water‐soluble gums, also called hydrocolloids, used in foods have been well documented. Most hydrocolloids are polysaccharides (gum Arabic, guar gum, carboxymethylcellulose, carrageenan, starch, and pectin) or proteins (such as gelatin). Hydrocolloids are widely used in the food industry in food systems for a variety of purposes, for example, as gelling agents, stabilizers, texture modifiers, and thickeners (Koochaki et al., 2008). Furthermore, numerous studies have shown that plant polysaccharides comprise biological features such as free radical scavenging and antivirus capabilities (Jooyandeh et al., 2018).

Alyssum is a genus of about 100–170 species of flowering plants in the family Brassicaceae, native to Egypt, Iran, Iraq, Arabia, and Pakistan. The seeds of this plant contain large amounts of mucilage. This mucilage is used in traditional medicine, especially in Iran as a traditional medicine (Nafchi et al., 2017).

Qodume Shirazi (*Alyssum homolocarpum*) seed mucilage (QSSM) has a great potential to be used as a new source of biodegradable film due to its suitable thickening/gelling action. The majority of QSSM is carbohydrate (85.33%) with small amount of uronic acid (5.63%). QSSM has low molecular weight (3.66 × 10^5^ Da) with relatively flexible chain and medium intrinsic viscosity (18.34 dl/g) at 25°C. The major sugar compositions of QSSM are galactose (82.97%), glucose (5.7%), rhamnose (5.04%), xylose (2.72%), mannose (3.04%), and arabinose (0.53%), and it is likely a galactan‐type polysaccharide. QSSM behaves like a typical polyelectrolyte because of the presence of carboxyl and hydroxyl groups. Mucilage extracted from Qodume Shirazi seed can be used as thickening, fat replacer, and stabilizer agent (Monjazeb Marvdashti et al., 2019).

The essential oils (Eos) in plants are recognized as safe (GRAS) and are among the most important natural products extracted from a variety of plants. Because of high antimicrobial and antioxidant properties, EOs are frequently used in the food industry as a flavoring, antioxidant, and antibacterial agent ( Gharibzahedi & Mohammadnabi, 2017).

There are about 8,000 herbal species in Iran, of which 2,300 are aromatic and medicinal herbs and are used in traditional medicine. Lavender is one of the herbs used in traditional Iranian medicine. Lavender is one of the most widely used medicinal plants in Iran. Lavender has been used in traditional medicine until now. This plant is from the genus Lavandula and from the mint family. This plant has 39 species (Palá‐Paúl et al., 2004). In Iran, there are two perennial species, *Lavandula sublepidota* and *Lavandula stricta*, which the former is an exclusive Iranian species.

Various studies have shown that when antimicrobial agents such as herbal extracts are added to films or edible coatings, they are slowly released to the food surface. So, they stay longer on food. In addition, oxidation can be effectively reduced by selecting suitable coatings that have low oxygen permeability (Sayyad et al., 2016).

In recent years, successful studies have been conducted on the application of various types of polysaccharide edible coatings containing different essential oils in extending the shelf life of different types of meat. Among such coatings, *Lallemantia iberica* mucilage containing *Cuminum cyminum* essential oil (Alizadeh Behbahani et al., 2020), *Salvia macrosiphon* mucilage in combination with *Myristica fragrans* (Kiarsi et al., 2020), the mixture of *Lepidium sativum* and *Heracleum lasiopetalum* (Barzegar et al., 2020), *Lallemantia royleana* mucilage blended with *Allium hirtifolium* (Alizadeh Behbahani & Imani Fooladi, 2018a), and *Plantago major* mucilage combined with *Anethum graveolens* essential oil (Alizadeh Behbahani et al., 2017) could be pointed out.

Therefore, the objectives of the present research were to produce bioactive edible coating based on QSSM loaded with different concentrations of lavender essential oil (LO) and evaluate the antioxidant and the antimicrobial activities of these substances in vitro and their effect on the quality and shelf life of ostrich meat during cold storage at 4°C.

## 2 MATERIALS AND METHODS

### 2.1 Materials

Qodume Shirazi seed (*Alyssum homolocarpum*) mucilage (QSSM) and *Lavandula sublepidota* L were purchased in Mashhad, Iran. Ostrich meat was purchased from local markets in Ahwaz, Iran. The microbial media, including Sabouraud dextrose agar (SDA), plate count agar (PCA), eosin methylene blue (EMB), violet red bile agar (VRBA), and mannitol salt agar (MSA), were supplied from Merck Co. All other chemicals and reagents used in this study were of analytical grade.

### 2.2 Extraction of the *Lavandula stoechas* essential oil and determination of chemical composition

Hydrodistillation method using a Clevenger‐type apparatus (power: 335 W; duration: 3 hr) was utilized to extract the essential oil from lavender. Quantification of LO components was performed using a Beifen 3420A gas chromatograph (Agilent Technologies 7890 A) equipped with a split injector, a flame ionization detector, and a DB‐5 capillary column (30 m × 0.25 mm, 0.25 μm stationary phase thickness). To identify the LO constituents, the obtained retention profile was compared with that of known samples already analyzed by a gas chromatograph (Agilent 7890A) coupled to a mass spectrometer (Agilent 5975C) with similar column and operating conditions (Alizadeh Behbahani & Imani Fooladi, 2018b; Kiarsi et al., 2020).

### 2.3 Determination of total phenolic content

In this experiment, 20 µl of LO at a concentration of 10 g/L was mixed with 2 ml of distilled water and 100 µl of folic acid reagent. After 3 min, 300 μl of sodium carbonate (Na_2_Co3) solution was added to them, and the solution was stirred for 2 hr, and finally, the absorbance of the solution was measured at 765 nm by spectrophotometer. The amount of the total phenolic content was determined as mg of gallic acid equivalent (GAE) by means of gallic acid calibration curve. Total phenolic content was reported as mg of gallic acid per gram (Alizadeh Behbahani et al., 2019a, 2019b).

### 2.4 Determination of total flavonoid content

Aluminum chloride colorimetric method was used to determine the number of flavonoids. LO in 0.5 ml (1:10 g/ml) and 1.5 ml methanol, 0.1 ml aluminum chloride (10% methanol), and 0.1 ml potassium acetate with 2.8 ml water distillate was combined. The solution was then incubated at room temperature for 30 min. The absorbance of each reaction compound was measured at 415 nm with a spectrophotometer (Alizadeh Behbahani et al., 2019a; Noshad et al., 2018). The total flavonoid content was expressed as mg quercetin equivalents (QE).

### 2.5 DPPH free radical scavenging assay

Scavenging activity was performed according to Kiarsi et al. (2020) method. For this purpose, 25 mg of LO was dissolved in 5 ml of distilled water; then, 0.1 ml of this solution was mixed with 3.9 ml of methanol DPPH solution (0.1 M methanol DPPH solution). The samples were then incubated for 60 min in a dark place at room temperature; the absorbance of the samples was measured by spectrophotometer against pure methanol at 517 nm. Pure methanol was used to zero the device. Free radical scavenging activity of the samples was calculated as percentage of inhibition (RSA) using Equation (1):

(1) $$\text{Inhibition}\;\text{of}\;\text{DDPH}\;\left(\%\right)=100\times \left(A_{1}‐A_{0}\right)/A_{0}$$

where *A*_0_ and *A*_1_ are the absorbance of the control and sample, respectively. Ascorbic acid was used as a positive control.

### 2.6 Chemical analysis of gum and ostrich meat

The moisture, ash, crude protein, and fat contents of the QSSM and ostrich meat were determined according to AOAC (2005).

### 2.7 Preparation of edible coating and treatment of ostrich meat samples

Two factors QSSM and LO as the central composition of active edible coatings were designed. For this aim, 2 g of the QSSM was mixed with 1 ml of Tween‐80 and made up to 100 ml with distilled water and heated and agitated using a magnetic stirrer. The LO was added to QSSM solution at levels of 0%, 0.5%, 1%, 1.5%, and 2% v/v. After that, 1 group out of the 5 groups of the samples was coated via being immersed in the QSSM solution for 1 min and the other groups were immersed in the QSSM solutions containing different concentrations of LO for the same period of time. A group that remained uncoated was considered as the control group (Alizadeh Behbahani et al., 2017).

### 2.8 Microbiological analysis

Ostrich meat samples were taken for microbial analysis on days 0 (after dipping treatment), 3, 6, and 9 days of refrigerated storage. Microbial culture was performed according to Alizadeh Behbahani and Imani Fooladi (2018b). For this purpose, each of the uncoated (control), QSSM, and QSSM + LO samples containing 0.5%, 1%, 1.5%, and 2% essential oil was thoroughly beaten by sterile Chinese mortar. Then, 5 g of the samples was removed and mixed with 45 ml of sterile physiological serum. The solutions were thoroughly mixed with vertex for 5 min and diluted to 10^–1^. Then, 1 ml of the solution was removed and mixed with 9 ml of sterile physiological serum and diluted to 10^–2^. Next, subsequent dilutions (10^–1^ to 10^–9^) were prepared in the test tubes. Dilutions were obtained for each sample separately. Then, 100 µl dilutions were removed and microbial counts were investigated by microbial culture according to the following methods:

1. Total viable count bacteria count in PCA (incubation at 37°C for 24 hr).
2. Psychrotrophic count in PCA (incubation at 20°C for 24 hr).
3. Mold and yeast count in SDA (incubation at 27°C for 72 hr).
4. *Escherichia coli* count in EMB (incubation at 37°C for 24 hr).
5. *Staphylococcus aureus* count in MSA (incubation at 37°C for 24 hr)
6. Coliform count in VRBA (incubation at 37°C for 24 hr).

Finally, all the plates were examined visually for the characteristics of a colony (shape, size, pigmentation, etc.) associated with each growth medium. Microbial colonies were counted and expressed as log10 CFU/g ostrich meat (Hamedi et al., 2017).

### 2.9 Physicochemical analysis

#### pH measurement

Minced ostrich meat samples (10 g) were blended with 90 ml of distilled water and homogenized. The pH values of sample were measured using a digital pH meter (Dragon Lab, MX‐S) at the 25°C (Amiri et al., 2019).

#### 2.9.1 Moisture content

The moisture contents (MC) of coated ostrich meat at 25°C were measured by drying the samples in an oven (Elektro Helios, 285 12, SE) at 102°C until constant weight (dry sample weight). Moisture content (%) was calculated using Equation (2):

(2) $$MC=\frac{m_{i}‐m_{d}}{m_{i}}\times 100$$

where *m*_i_ and *m*_d_ are initial and dried sample weights, respectively (Guerrero et al., 2015).

#### 2.9.2 Texture

Hardness of samples was measured based on Akwetey and Knipe (2012) method. The test was carried out by a computer‐connected probe, TA. XT plus, made by the UK's Stable Micro System. A probe with a diameter of 36 mm (36 p/36) was used for the experiment. In this test, compression force by a 10 kg weight was applied to the specimens (2 × 2 × 2 cm) up to 50% of its initial height at a constant velocity of 5 mm/s. The test was performed on days 0, 3, 6, and 9, and the samples were evaluated for tissue hardness parameter.

#### 2.9.3 Color

The color of the coated ostrich meat samples was measured by a Hunter Lab color meter (Konica Minolta, CR‐400, JP) and was reported by the CIE system. The colorimeter was calibrated with a white standard. The values of L*, a*, and b*, respectively, which represent the brightness of *L* from black (zero) to white (100), reds from green (negative values) to red (positive values), and yellow *b* from blue (negative values) to yellow (positive values), were measured. ∆*E* (overall color change) was also investigated (Tahmasebi‐Pour et al., 2014).

### 2.10 Sensory evaluation

The 9‐point hedonic method was used to evaluate the organoleptic properties of ostrich meat samples. For this purpose, the samples were evaluated in terms of color, aroma, and overall acceptability (1: dislike extremely to 9: like extremely) by 15 semitrained panelists. Six samples/treatments of ostrich meat were given to each panelist separately in small porcelain dishes. The panelists were not aware of the experimental approach, and the samples were blind‐coded. Samples that scored more than 4 were considered as valid samples (Alizadeh Behbahani et al., 2020; Nisar et al., 2019).

### 2.11 Statistical analysis and neural network modeling–genetic algorithm

All experiments were performed in at least triplicate and were analyzed by ANOVA using SPSS software (ver. 21, IBM Inc.). Duncan's multiple range tests with 95% confidence level were used to compare the means.

An artificial neural network (ANN) consisting of a set of interconnected neurons is able to estimate outputs based on input and data. The designed lattice type was multilayer perceptron (MLP) in which the input layer consisted of 2 neurons (LO concentration and shelf life) and the output layer contained 5 neurons (moisture content, color index, total microbial load, hardness, and pH). Levenberg–Marquardt (LM) training algorithms were used to update the ANN weights. This algorithm is one of the most commonly used algorithms because it makes network training very fast and minimizes the level of error. In fact, this algorithm is designed to increase network learning speed based on the Hessen matrix (Salehi & Razavi, 2012).

One of the problems that may occur when training a neural network is network learning. In this case, the error is acceptable when training the network, but when evaluating, the network error is much more than the error of the training data. There are two ways to avoid overlearning: 1—to stop training; 2—to choose the lowest number of neurons in the hidden layer. The second method was used in this study. In order to train the network, the data were first randomly divided into three parts, with 60% of the data used for training, 20% of the data used for evaluation, and 20% of the data used for network testing. During network training, the training process was interrupted when the error between training and evaluation data increased.

The number of neurons in the hidden layer was optimized by genetic algorithm method. The initial population was assumed to be 100 generations and maximum 5 generations. The probability of fusion and mutation was 0.9 and 0.01, and the number of neurons to optimize was 1 to 20, respectively. In order to predict the performance of the obtained networks, the parameters of mean squared error (*MSE*), mean absolute error (*MAE*), and correlation coefficient (*r*) were used to predict the investigated parameter. In order to investigate the relative importance of each network input in modifying the output factors of the model, the relative sensitivity of the output factors to the network input parameters was also evaluated.

## 3 RESULTS AND DISCUSSION

### 3.1 Chemical compositions of lavender essential oil (LO)

Analysis of essential oil from lavender was performed by GC/MS device. The results of this analysis showed the presence of 12 compounds in the essential oil, which accounted for a total of 99.41% of the essential oils. The most representative compounds of LO were monoterpene, and among them, the main constituents were linalool (43.3%), linalyl acetate (19.1%), β‐myrcene (11.61%), D‐limonene (5.43%), and 1,3,6‐octatriene (5.31%) (Table 1). The result of LO analysis was similar to other previous studies on the lavender species. These studies have also shown that LO is rich in linalool (Rapper et al., 2016; Dimitra et al., 2000; Falk et al., 2009; Morgan et al., 2006; Shaw et al., 2007). The difference in composition is probably due to local, climatic, seasonal, and experimental conditions (Alizadeh Behbahani et al., 2017).

**TABLE 1:** Chemical compositions of lavender essential oil (LO)

No	Compound	Percentage	KI^a^	Retention time (min)
1	β‐Myrcene	11.61	989	11.63
2	Limonene	5.43	1,031	13.52
3	1,3,6‐Octatriene	5.31	1,040	14.70
4	Linalool	43.3	1,098	17.38
5	Alloocimene	2.02	1,128	18.08
6	Camphor	1.46	1,143	18.45
7	Borneol	0.69	1,164	19.28
8	Terpinen−4‐ol	3.5	1,178	19.74
9	α‐Terpineol	0.77	1,187	20.21
10	Linalyl acetate	19.1	1,261	22.48
11	Octadien	3.66	1,278	25.46
12	Neryl acetate	2.56	1,364	26.00
Total		99.41%		

^a^ KI: the Kovats retention indices relative to C_8_‐C_20_ n‐alkanes were determined on DB5 capillary column.

### 3.2 Chemical compounds of Qodume Shirazi seed mucilage

The general chemical compositions of QSSM were measured, and the mean values were recorded as follows: moisture (4.5 ± 0.1), protein (3.5 ± 0.05), fat (2.8 ± 0.01), ash (5.3 ± 0.02), and carbohydrate (83.9 ± 0.03). According to results obtained by Anvari et al. (2015), the extracted mucilage is mostly comprised of carbohydrates (61.0 ± 0.6%) and proteins (17.91.0%). The difference in results is probably due to different plant growth conditions or different extraction methods.

### 3.3 Chemical compounds of ostrich meat

The proximate chemical analysis of the ostrich meat was determined as follows: moisture (75.01 ± 0.01), protein (22.96 ± 0.02), fat (0.9 ± 0.01), and ash (1.13 ± 0.09). The lower fat content along with the higher protein content is of particular interest of the ostrich meat. In fact, the fat/protein ratio definitely favors ostrich meat. The percentage of each ingredient was similar to those previously reported by Paleari et al. (1998) and Akram et al. (2019). The difference in the chemical composition of ostrich meat depends on several factors. Among them, nutrition, age, slaughter time, and environmental conditions are the most important factors which cause variations in different regions (Alizadeh Behbahani et al., 2017).

### 3.4 Antioxidant activity, total phenolic content, and total flavonoid content

The antioxidant activity, total phenolic content, and total flavonoid content of the LO were equal to 68.9%, 97.7 mg GAE/g, and 1.6 mg QE/g, respectively. Similar result with a little difference was reported by Spiridon et al. (2011); Dif et al. (2017); and Nurzynska‐ Wierdak and Zawislak (2016).

### 3.5 Microbiological analysis of ostrich meat

#### 3.5.1 Total viable count (TVC)

**FIGURE 1:**
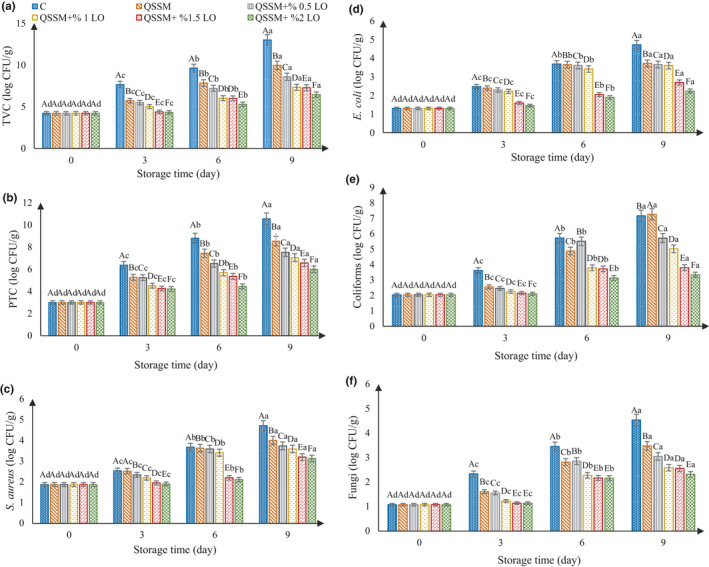
Changes in total viable count (a), psychrotrophic count (b), *S. aureus* count (c), *E. coli* count (d), coliforms count (e), and fungi count (f) of the ostrich meat samples stored at 4°C for 9 days

The TVC of ostrich meat samples coated with QSSM incorporated with LO (0%, 0.5%, 1%, 1.5%, and 2%) during 9 days of storage at refrigerated temperature is shown in Figure 1a. The results revealed that the microbial count of all examined groups inclined to increase gradually with storage period. However, the increase in TVC in the samples coated with QSSM + LO was lower than the control sample. With the increase in essential oil, this slope was also decreased. The TVC of the control sample on the 3 days was higher than the standard, indicating that the duration of ostrich meat storage in the refrigerator (4°C) was maximum 3 days. The results showed that the coating of ostrich meat increased the meat shelf life. According to these results, the shelf life of coated ostrich meat containing 2% LO was 6 days at 4°C as compared to the control sample. The pH value of ostrich meat is one of the determinant factors of TVC. The pH of the ostrich meat is near to 6 and is therefore heavily influenced by microbial growth (Van et al., 2005). Seydim et al. (2006) reported that addition of rosemary extract and sodium lactate to ostrich meat can reduce TVC. However, the use of a mixture of sodium lactate and rosemary extract provided better antimicrobial results than when they used alone. Therefore, sodium lactate alone or in combination with rosemary extract can be used to reduce microbial growth and increase the shelf life of minced meat when stored in the refrigerator.

#### 3.5.2 Psychrotrophic count

The results of statistical analysis showed that the treatments and storage time had a statistically significant effect on psychrotrophic bacterial count in ostrich meat (*p* ≤ .05). Our results are shown in Figure 1b. The initial PTC of samples was 3 log colony‐forming unit (CFU)/g and increased in all samples. The highest and the lowest number of psychrotrophic bacterial during the cold storage were observed in the control and the sample coated with QSSM and 2% LO, respectively. The coating of the sample with QSSM and LO had positive effects on preservation of the ostrich meat due to reduction of oxygen exchange. Pseudomonas species are unable to survive in the absence of oxygen because they are extremely aerobic (Alghooneh et al., 2015; Alizadeh Behbahani et al., 2017).

Bingol and Ergun (2011) examined the effect of modified atmosphere containing different amounts of oxygen, carbon dioxide, and nitrogen on the chemical and microbial properties of ostrich meat. The researchers found that the use of modified atmosphere rather than the use of conventional air conditions reduced the number of bacterial cells during 7‐day storage period. *Pseudomonas aeruginosa* species are highly aerobic and oxygen‐dependent, and oxygen deficiency prevents the growth of these bacteria.

#### 3.5.3 *Staphylococcus aureus* count

The results of variance of all treatment data showed that the *S. aureus* count increased significantly (*p* ≤ .05) during storage time (Figure 1c). The increase in microbial load in the samples containing the coating increased with a slower gradient. Djenane et al. (2012) investigated the effects of lavender and peppermint essential oil on *S. aureus* and *Escherichia coli* count in minced beef. Their results showed that both essential oils were able to prevent the growth of *S. aureus* and *E. coli* in minced meat samples compared to the control sample.

#### 3.5.4 *Escherichia coli* count

The results of *E. coli* count during storage are illustrated in Figure 1d. The results showed that the initial level of *E. coli* in all samples was 1.3 log CFU/g and the count enhanced with increase in the storage time. Adaszyńska‐Skwirzyńska and Szczerbińska (2018) investigated the antimicrobial effect of LO on *E. coli* and *S. aureus*. Their results showed that the essential oil of lavender had a significant antimicrobial effect on both bacteria, but the antimicrobial effect of the essential oil on *E. coli* was less than that on *S. aureus* because of its cell wall. Alizadeh Behbahani et al. (2017) reported that the use of *Plantago major* seed mucilage as a novel edible coating incorporated with *Anethum graveolens* essential oil significantly reduced the number of *E. coli* and *S. aureus* during storage (18 days) in the treated samples.

#### 3.5.5 Coliform count

The results of counting the total number of ostrich meat coliform bacteria maintained at 4°C are given in Figure 1e. The initial bacterial coliform count was 2.04 log CFU/g. The results showed that the number of coliform bacteria increased during the storage time in all treatments, but on day 9 of storage, there was a significant difference between the control sample (7.17 log CFU/g) with the samples containing 1.5% essential oil (3.8 log CFU/g) and the samples containing 2% essential oil (3.34 log CFU/g). The results of research by Seydim et al. (2006) showed that using vacuum packaging containing rosemary essential oil and sodium lactate and their mixture, the number of coliform bacteria of ostrich meat reduced as compared to the control sample during the storage time at 3°C. Jebelli Javan et al. (2018) investigated the effect of chitosan and *Trachyspermum ammi* essential oil on microbial growth, proteolytic spoilage, lipid oxidation, and sensory attributes of chicken fillet during refrigerated storage for 12 days. The results showed that the use of chitosan edible coating containing *Trachyspermum ammi* essential oil significantly reduced coliform bacteria as compared to the control sample. But the number of coliform bacteria in the coated sample without essential oil was not significantly different from the control sample.

#### 3.5.6 Mold and yeast count

The results of the counts of molds and yeasts in ostrich meat stored at 4°C are shown in Figure 1f. The results showed that the initial level of fungi in the samples was 1.08 log CFU/g. Over time, the count increased in all treatments. The fungi are formed on the sample surface due to being aerobic. The presence of QSSM + LO reduced the growth of fungi due to the prevention of oxygen exchange (Heidari et al., 2015). Microbial load was significantly (*p* ≤ .05) different in all days and in different treatments. Hamedi et al. (2017) investigated the antimicrobial effect of edible coating of sodium alginate and galbanum gum with *Ziziphora persica* essential oil on shelf life of chicken fillets at 4°C. The results showed that edible coating significantly reduced the number of molds and yeast in the coated samples compared to the control sample at 12 days. Noori et al. (2018) investigated the antimicrobial effect of edible coating of sodium caseinate nano‐emulsion containing ginger essential oil on poultry meat quality at 4°C. Their results showed a significant decrease in the number of molds and yeasts in the coated samples as compared to the control samples during 12‐day storage. Alizadeh Behbahani and Imani Fooladi (2018b) reported a significant decrease in the number of molds and yeasts in beef samples coated with Balangu seed mucilage and Feverfew essential oil compared to control samples during 18 days of refrigerated sample storage.

### 3.6 Physicochemical analysis

#### 3.6.1 pH measurement, moisture content, and texture changes

**FIGURE 2:**
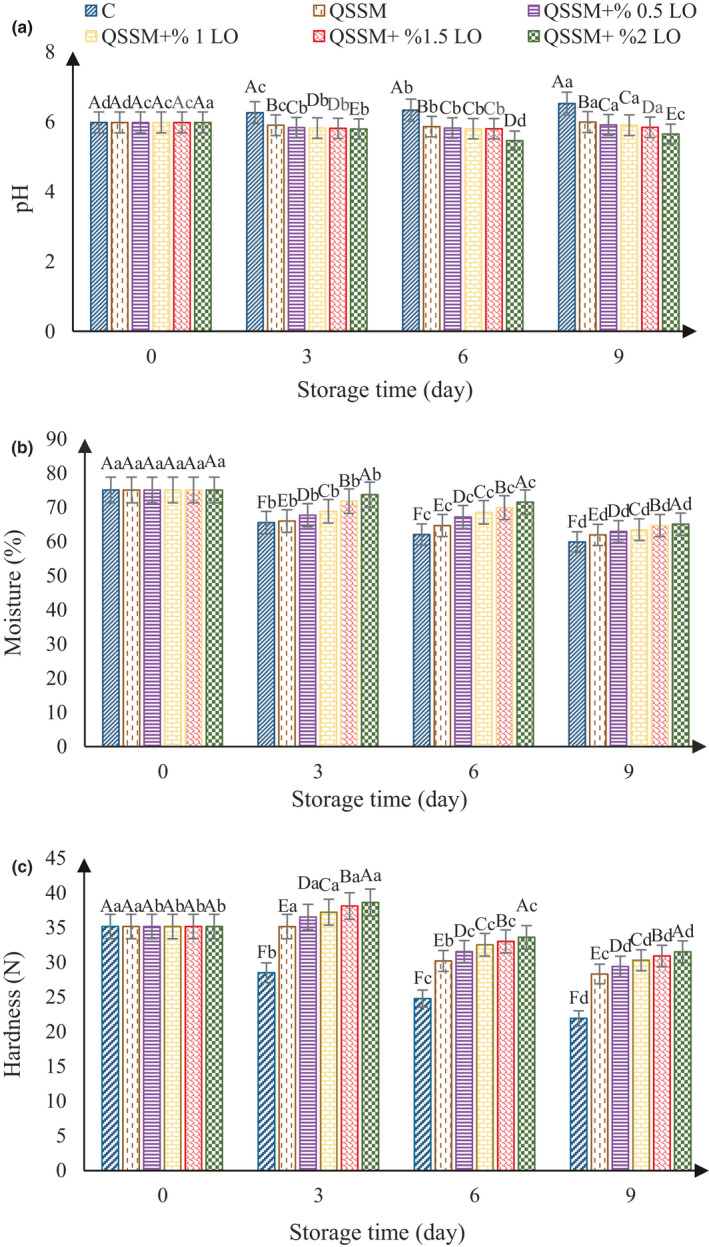
Effect of different concentrations of lavender essential oil (LO) added to Qodume Shirazi seed mucilage (QSSM)‐based edible coating on pH (a), moisture (b), and tissue (c) of ostrich meat during 9 days of storage at 4°C

The results of pH changes of ostrich meat samples stored at 4°C are shown in Figure 2a. The initial pH of the samples was 5.97. Results of analysis of variance showed that pH of control sample increased during the storage. The reason for the increase in pH is the destruction of the tissue of the meat sample by microorganisms, which causes the breakdown of protein constituents and the production of nitrogenous compounds and ultimately increases the pH. In the QSSM‐coated samples and the QSSM + LO‐coated samples, due to the positive effects of the coating on microbial growth, the pH of the samples decreased at days 3 and 6 and then increased at day 9. Various studies have shown that microbial activity causes carbon dioxide production; some of the essential oils and extracts in film and edible coatings can reduce or increase the permeability to carbon dioxide (Bifani et al., 2007). It seems that the LO in the QSSM has reduced permeability, resulting in increased carbon dioxide content in the coating, and ultimately increased carbon dioxide, reducing the pH of the samples. On the other hand, increased carbon dioxide can also reduce microbial growth. In the QSSM‐coated samples, due to the absence of essential oil, the permeability was higher than that of the QSSM + LO‐coated samples, which resulted in lower pH on days 3 and 6 and a higher pH on day 9 as compared to the QSSM + LO samples. Generally, during the storage time, the pH decreased on days 3 and 6 in the coated samples with increasing essential oil content. Significant difference (*p* ≤ .05) in the pH was observed in all samples at different levels of QSSM and QSSM + LO on the same days and in the same samples on different days.

Carolina Pelaes Vital et al. (2016) studied the effect of edible and active coating (with rosemary and oregano essential oils) on beef characteristics and consumer acceptability. The results showed that the pH of the control samples increased during shelf life, but the pH of the coated samples without essential oils and coated samples containing rosemary and oregano essential oils decreased and then increased. Alizadeh Behbahani and Imani Fooladi (2018b) investigated the effect of edible coating of Balangu seed mucilage and Feverfew essential oil on the shelf life of beef slices during refrigerated storage. The results showed that the pH of the control samples increased during storage due to microbial activity and production of nitrogen compounds, but the pH of the coated samples without essential oils and coated samples containing chamomile essential oil decreased during the storage period due to a decrease in carbon dioxide permeability.

The moisture content of ostrich meat stored at 4°C is shown in Figure 2b. The results showed that the initial moisture content of the samples was 75%. Over time, the moisture content of all samples decreased, but the rate of moisture content decreased in the coated samples. Also, the results of data analysis of variance showed that all samples had significant differences in the moisture content (*p* ≤ .05) on day 9. Previous research has shown that water storage capacity has a direct relationship with pH; as pH decreases, water storage capacity decreases. Therefore, one of the reasons for the decrease in the moisture content in the samples is likely to be a decrease in water storage capacity (Botha et al., 2006). On the other hand, Nisar et al. (2019) stated that addition of essential oil to edible coating increased the ability to prevent moisture loss. In fact, water binds to the hydrophilic portions of the coating to exit the coating; the addition of the essential oil to the coating increases the hydrophobic part of the coating matrix, thereby preventing further physical barriers to moisture transfer, ultimately reducing its water transmission.

The results of texture evaluation of the ostrich meat maintained at 4°C are presented in Figure 2c. The results of analysis of variance showed that the hardness of all samples decreased with passing time. On day 9, the hardness of all samples showed a significant difference (*p* ≤ .05). In the QSSM + LO samples, the hardness increased on day 3 of the storage and then decreased on days 6 and 9. No significant difference (*p* ≤ .05) was observed in the QSSM samples on days 1 and 3, but on days 6 and 9, the hardness decreased (Figure 2c).

The results of this study were in line with the findings of Rahnemoon et al. (2018). These researchers investigated the effect of alginate coating containing pomegranate peel extract on shelf life, texture, and color characteristics of chicken breast meat. The researchers attributed the decrease in hardness of the samples to the destruction of the meat tissue by microorganisms. On the other hand, with decreasing water‐holding capacity due to lower pH in the QSSM and LO samples, the hardness of the samples increased and then decreased. Lowering the pH reduces water storage capacity. Therefore, the hardness was increased on day 3. On day 6, due to tissue damage by microorganisms, the hardness decreased despite lower pH. On day 9, due to increases in pH and tissue destruction by microorganisms, the rate of hardness decreased (Lawrie & Ledward, 2006).

#### 3.6.2 Color measurement

**FIGURE 3:**
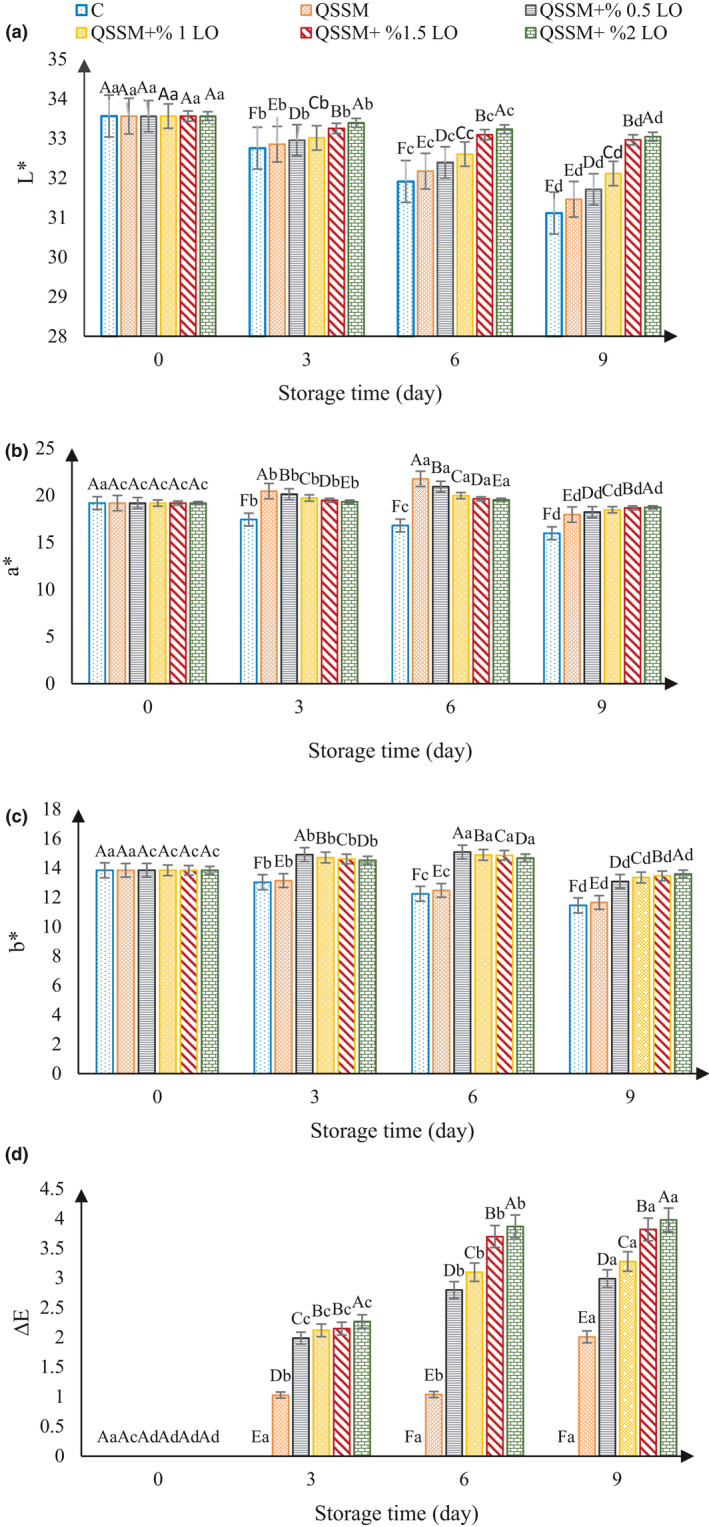
Effect of different concentrations of lavender essential oil (LO) added to Qodume Shirazi seed mucilage (QSSM)‐based edible coating on color values (L* (a), a* (b), b* (c), and Δ*E* (d)) of ostrich meat during 9 days of storage at 4°C

The results of color evaluation of the ostrich meat samples stored at 4°C during 9 days of storage are presented in Figure 3a–d. The results showed that during the storage period, the amount of L* or brightness decreased in all treatments, but the slope of the diagram was milder in the coated samples than in the control sample (Figure 3a). The results showed that a* factor, which measures the redness of the sample, was increased and then decreased in the coated samples. The level of this factor at day 9 of the storage in all samples was significantly different at the level of (*p* ≤ .05), but the decrease in sample containing 2% LO was lower than in other samples (Figure 3b). The results showed that the b* value, which is related to the yellowness of the sample, decreased during the storage in the control and QSSM‐coated samples, but in the QSSM + LO samples, it initially increased and thereafter decreased (*p* ≤ .05). Changes in the sample containing 2% essential oil were lower than in the other samples during the storage time (Figure 3c). The results showed that the Δ*E* factor, which reflects the overall color change of the sample and is dependent on the L*, a*, and b* factors, increased during the storage time (Figure 3d).

Van et al. (2005) reported that increasing pH in ostrich meat darkens the meat color. Vital et al. (2016) investigated the effect of active edible alginate coating with rosemary and oregano essential oils on beef. The results of these researchers are consistent with the present study. These researchers attributed the decrease in factor L* during storage to changes in protein structure due to oxidation that may increase light scattering, and in the samples containing the coating, the reduction was less due to partial inhibition of oxygen exchange. Adding essential oils to the samples due to their antimicrobial properties slowed down the microbial activity, resulting in lower protein degradation rate and lower brightness than the control and QSSM samples.

The researchers also reported that in fresh meat, when cutting any kind of meat, the so‐called meat color flowers and oxygenated myoglobin pigment produce oxymyoglobin; it is brown in color and reduces the sample red color during storage. In the coated samples up to 6 days of the storage, due to the coating and reduction of oxidation process, the amount of oxymyoglobin was higher and this resulted in redness escalation but it decreased after 6 days of storage. The essential oil also reduced the rate of oxidation due to its antioxidant substances and the slope of the redness curve decreased with increasing essential oil content.

**FIGURE 4:**
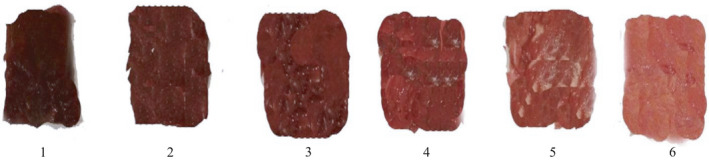
Effect of different concentrations of lavender essential oil (LO) added to Qodume Shirazi seed mucilage (QSSM)‐based edible coating on color changes of ostrich meat during 9 days of storage at 4°C (1: control; 2: QSSM + 0% LO; 3: QSSM+%0.5 LO; 4: QSSM+ %1 LO; 5: QSSM+%1.5 LO; and 6: QSSM+%2 LO)

Figure 4 shows the color changes of the control ostrich meat and treated samples after 9 days of storage.

### 3.7 Sensory evaluation

**FIGURE 5:**
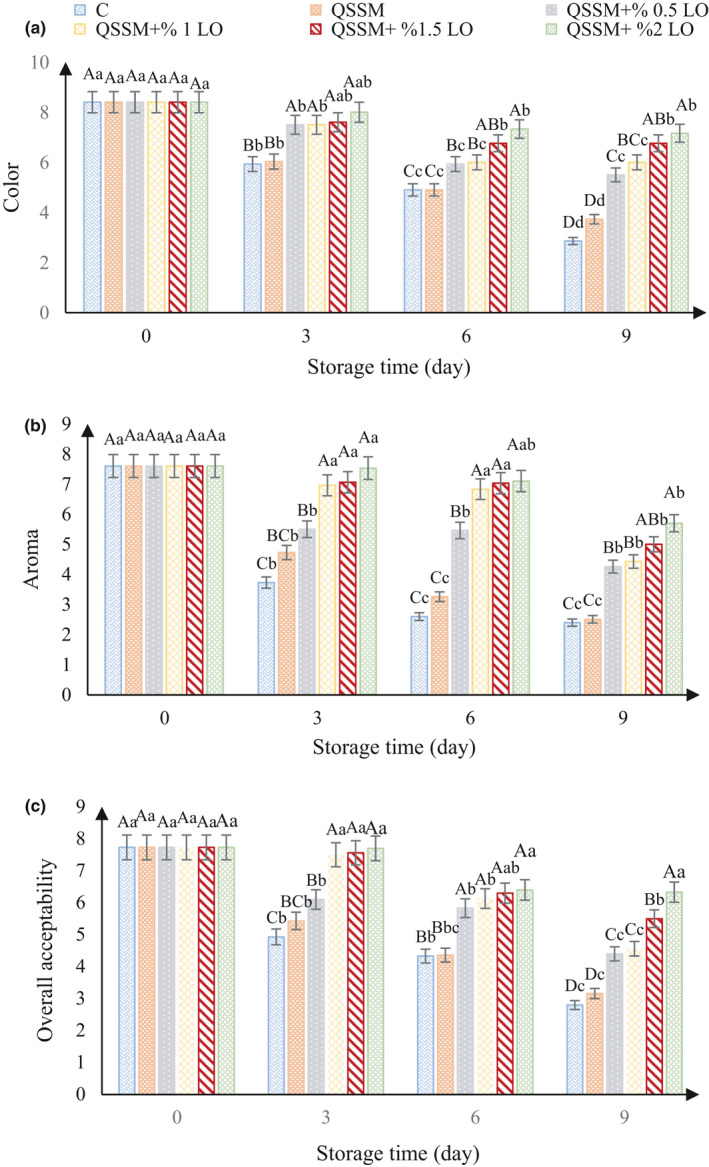
Effect of different concentrations of lavender essential oil (LO) added to Qodume Shirazi seed mucilage (QSSM)‐based edible coating on sensory attributes (color (a), aroma (b), and overall acceptability (c)) of ostrich meat during 9 days of storage at 4°C

The results of sensory evaluation (color, aroma, and overall acceptability) of ostrich meat samples kept at 4°C are shown in Figure 5a–c. In this way, ostrich meat samples with a score above 4 can be accepted (Hansen et al., 1995; Nisar et al., 2019). Sensory scores (color) less than 4 were observed for control and QSSM samples on day 9. For the QSSM + LO samples, the retention score by day 9 was greater than 4 (Figure 5a).

The results of analysis of variance showed that in sensory evaluation of samples (aroma), score less than 4 for control sample was observed on day 3. For the QSSM sample, score less than 4 was obtained on day 6, and for QSSM + LO samples containing 0.5%, 1%, 1.5%, and 2% essential oil up to day 9, a score above 4 was obtained (Figure 5b). Various studies have shown that *Pseudomonas* spp. causes spoilage in unpackaged beef samples (Alizadeh Behbahani et al., 2017; Dainty et al., 1989).

On the basis of sensory tests, the ostrich meat control, the QSSM, and the QSSM + LO samples had (overall acceptability) score above 4 on days 6, 6, and 9, respectively (Figure 5c).

Comparison between the results of microbial count and sensory (overall acceptability) of the samples showed that there is a good correlation between the data obtained from these tests. For the sample containing 2% essential oil, the shelf life of microbial tests (total microbial load count) and sensory characteristics (overall acceptability) were appropriate until day 9 of storage. For the control sample, microbial load was adequate up to day 3 and overall acceptability was adequate up to day 6.

Fazlara et al. (2017) investigated the effect of using gelatin–Avishan Shirazi (*Zataria multiflora Bioss*) coating on microbial, chemical, and sensorial characteristics of ostrich fillets in refrigerated conditions. These researchers showed that the control sample had appropriate overall acceptability, microbial load, and physicochemical characteristics up to 3 days. Regarding the gelatin coating without thyme, it was also noted that it retains usability for up to 3 days in terms of microbial load and up to 6 days in terms of physical and sensory changes. For Shiraz thyme treatment alone, they reported acceptability for microbial load up to 6 days and for physical changes up to 9 days, while for gelatin‐containing thyme treatment, they found appropriate count for psychrophilic up to 6 days, mesophilic up to 12 days, and sensory and physical changes up to 12 days.

### 3.8 Neural network modeling–genetic algorithm

The optimal structure of the artificial neural network (ANN) was obtained to model the ostrich meat coating process during trial and error. The best artificial ANN structure and related parameters are shown in Table 2. The Levenberg–Marquardt (LM) algorithm, one of the most commonly used training algorithms, was used to update ANN weights. This algorithm performs very fast network training and minimizes the level of error. In fact, this algorithm was designed to speed up network learning.

**TABLE 2:** The best artificial neural network (ANN) structure for modeling the meat coating process

Multilayer perception	Type
Tan Axon	Transfer
Levenberg–Marquardt	Learning rule
1,000	Learning period
1	Number of hidden layers
10	The number of neurons in the hidden layer
The lowest mean squared error (*MSE*)	Measurement criteria

The performance of the network in predicting the microbial and qualitative properties of the coated samples for the test data is shown in Table 3. Low *MSE* indicates the ability of the network to predict changes in this parameter.

**TABLE 3:** Performance of genetic algorithm optimized neural network for predicting qualitative and microbial properties of the ostrich meat samples without and with QSSM + LO during storage at 4°C for 9 days

Performance	Moisture	pH	Chroma	TVC	Hardness
*MSE*	0.113084114	0.002593513	0.940061989	0.062692623	0.053105265
*NMSE*	0.005198725	0.133667841	0.4215096	0.01989906	0.006973125
*MAE*	0.301636668	0.031777163	0.431425678	0.196148593	0.193794808
Min Abs Error	0.098656529	0.001386638	0.013294699	0.007703981	0.084053627
Max Abs Error	0.52772656	0.169172552	3.593463816	0.550806149	0.481981193
*r*	.997630079	.938340014	.768659774	.99033706	.997042814

**FIGURE 6:**
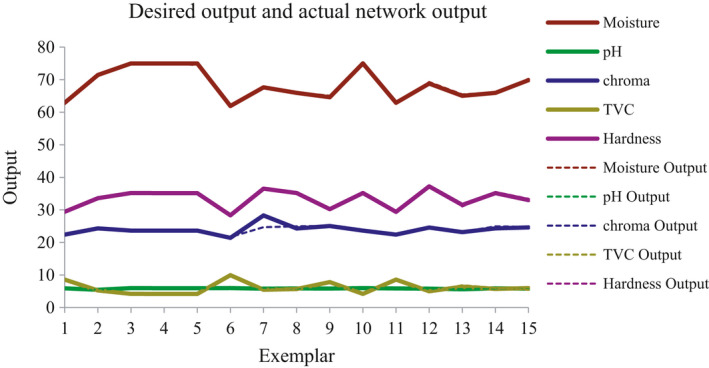
Real and predicted results by neural network optimized with genetic algorithm

The results showed that neural network with 10 neurons in hidden layer had the lowest mean squared error (*MSE*) and mean absolute error (*MAE*) and the highest correlation coefficient (*r*) for predicting the quality and microbial properties of coated meat during storage time. The efficiency of the optimal ANN in predicting the qualitative and microbial properties of the coated meat is shown in Figure 6. In this figure, the actual values of the values obtained from the experiments are shown against the values predicted by the model. High correlation coefficients indicated high performance of ANN in predicting quality and microbial properties of coated meats.

**FIGURE 7:**
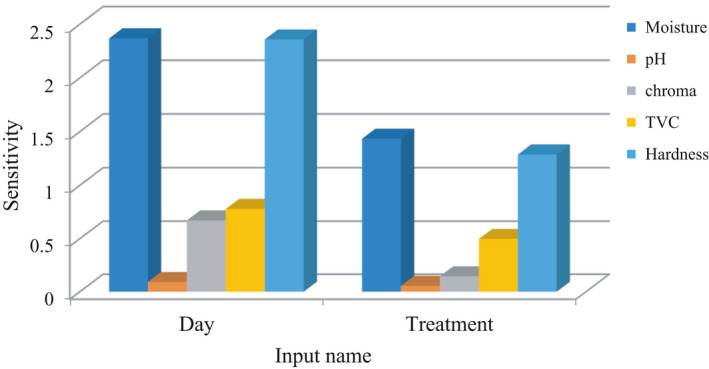
Sensitivity analysis results attained by optimal neural network

In order to investigate the effect of input parameters and identify the most effective factor, the sensitivity analysis test was performed on the optimal network. The results showed that the shelf life was more effective in predicting the quality and microbial properties than the essential oil concentration in the coatings (Figure 7). The results of this study were similar to those of Sagdic et al. (2012); Fernández et al. (2010); Yolmeh et al. (2014); Alizadeh Behbahani and Imani Fooladi. (2018b).

## 4 CONCLUSION

The incorporation of LO to a natural hydrocolloid, QSSM, fabricated an active edible coating for possible food packaging applications. The lightness (L* value) and hardness of all the ostrich meat samples were reduced during storage. From a microbiological point of view, the cold storage time for control sample and sample coated without essential oil was 3 days, and for 0.5%, 1%, 1.5%, and 2% essential oils, it was 3, 3, 6, and 9 days, respectively. The coating containing 2% LO conferred good quality characteristics to the ostrich meat and expanded its refrigeration shelf life. The results showed that neural network with 10 neurons in hidden layer had the lowest mean squared error (*MSE*) and mean absolute error (*MAE*) and the highest correlation coefficient (*r*) for predicting the quality and microbial properties of coated meat during storage time.

## CONFLICT OF INTEREST

The authors declare no conflict of interest.

## ETHICAL APPROVAL

The authors declare that this study did not involve human or animal subjects, and human and animal testing were unnecessary in this study.
